# α-MSH-induced activation of spinal MC1R but not MC4R enhances colorectal motility in anaesthetised rats

**DOI:** 10.1038/s41598-020-80020-x

**Published:** 2021-01-12

**Authors:** Hiromi H. Ueda, Kiyotada Naitou, Hiroyuki Nakamori, Kazuhiro Horii, Takahiko Shiina, Tatsunori Masatani, Mitsuya Shiraishi, Yasutake Shimizu

**Affiliations:** 1 Department of Basic Veterinary Science, Joint Faculty of Veterinary Medicine, Kagoshima University, 1-21-24 Korimoto, Kagoshima, 890-0065 Japan; 2 Department of Cell Physiology, Nagoya City University Graduate School of Medical Sciences, 1 Kawasumi, Mizuho-cho, Mizuho-ku, Nagoya, 467-8601 Japan; 3 Laboratory of Physiology, Department of Basic Veterinary Science, The United Graduate School of Veterinary Sciences, Gifu University, 1-1 Yanagido, Gifu, 501-1193 Japan; 4 Transboundary Animal Diseases Research Center, Joint Faculty of Veterinary Medicine, Kagoshima University, Kagoshima, 890-0065 Japan; 5 Center for Highly Advanced Integration of Nano and Life Sciences, Gifu University (G-CHAIN), Gifu, Japan

**Keywords:** Neurophysiology, Spine regulation and structure, Gastrointestinal diseases, Gastrointestinal models, Gastrointestinal system

## Abstract

The central nervous system is involved in regulation of defaecation. It is generally considered that supraspinal regions control the spinal defaecation centre. However, signal transmission from supraspinal regions to the spinal defaecation centre is still unclear. In this study, we investigated the regulatory role of an anorexigenic neuropeptide, α-MSH, in the spinal defaecation centre in rats. Intrathecal administration of α-MSH to the L6-S1 spinal cord enhanced colorectal motility. The prokinetic effect of α-MSH was abolished by severing the pelvic nerves. In contrast, severing the colonic nerves or thoracic cord transection at the T4 level had no impact on the effect of α-MSH. RT-PCR analysis revealed MC1R mRNA and MC4R mRNA expression in the L6-S1 spinal cord. Intrathecally administered MC1R agonists, BMS470539 and SHU9119, mimicked the α-MSH effect, but a MC4R agonist, THIQ, had no effect. These results demonstrate that α-MSH binds to MC1R in the spinal defaecation centre and activates pelvic nerves, leading to enhancement of colorectal motility. This is, to our knowledge, the first report showing the functional role of α-MSH in the spinal cord. In conclusion, our findings suggest that α-MSH is a candidate for a neurotransmitter from supraspinal regions to the spinal defaecation centre.

## Introduction

Defaecation is regulated by the enteric nervous system in the periphery and also by the central nervous system. The enteric nervous system is capable of operating independently of the central nervous system, and the mechanisms by which it regulates motility have thus been extensively studied by using isolated preparations^1^. On the other hand, mechanisms by which colorectal motility is controlled by the central nervous system are not fully understood. Available evidence has suggested that there are two defaecation centres, *i.e.*, the supraspinal defaecation centre and spinal defaecation centre, in the central nervous system^2^. The supraspinal defaecation centre activates or inhibits the spinal defaecation centre, and the spinal defaecation centre modulates the enteric nervous system^2^. In addition, emerging evidence has indicated that monoamines including noradrenaline, dopamine and serotonin are involved in signal transmission from supraspinal regions to the spinal defaecation centre^3–8^. Peptides including corticotropin-releasing hormone act in brain sites and influence colonic motor function^9,10^. Furthermore, ghrelin and somatostatin have also been shown to activate the spinal defaecation centre^11,12^, although their physiological significance remains to be elucidated.

It has been shown that ghrelin receptors are expressed in the spinal defaecation centre and that activation of the receptors enhances colorectal motility by activating preganglionic neurons of the sacral parasympathetic nuclei innervating the colorectum^13,14^. In this study, we focused on the action of α-melanocyte-stimulating hormone (α-MSH) in the spinal defaecation centre. α-MSH is an anorexigenic neuropeptide derived from the precursor polypeptide proopiomelanocortin (POMC) and it binds to melanocortin receptors (MCR) subtypes 1, 3, 4 and 5^15^. The peptide is abundantly expressed in the arcuate nucleus (ARC) of the hypothalamus^16^. The ARC is a key region regulating energy homeostasis and is composed of two types of neurons, orexigenic neuropeptide Y (NPY)-containing neurons and anorexigenic POMC-containing neurons, both of which project to the paraventricular nucleus (PVN)^17^. Ghrelin activates NPY neurons and causes food intake^18,19^. POMC neurons are activated by glucose or insulin^20,21^, and activation of POMC neurons leads to inhibition of food intake^15,22^. Therefore, α-MSH has the opposite effect to that of ghrelin on regulation of food intake in the hypothalamus.

In the spinal cord, it is thought that α-MSH is released from POMC-containing axons projecting from the ARC^23–25^ and MC4R is expressed^26–28^. Owing to the fact that α-MSH has the opposite effect to that of ghrelin in the hypothalamus, we assumed that α-MSH also exerts the opposite effect to the prokinetic action of ghrelin on colorectal motility in the spinal defaecation centre. To verify this hypothesis, we examined the effects of α-MSH administration into the L6-S1 spinal cord, where the defaecation centre is located, in anaesthetised rats. Interestingly, intrathecally administered α-MSH enhanced colorectal motility, contrary to our assumption. Therefore, in this study, we performed further investigation into the mechanisms of α-MSH-induced enhancement of colorectal motility.

## Results

### Effect of intrathecally administered α-MSH on colorectal motility

**Figure 1:**
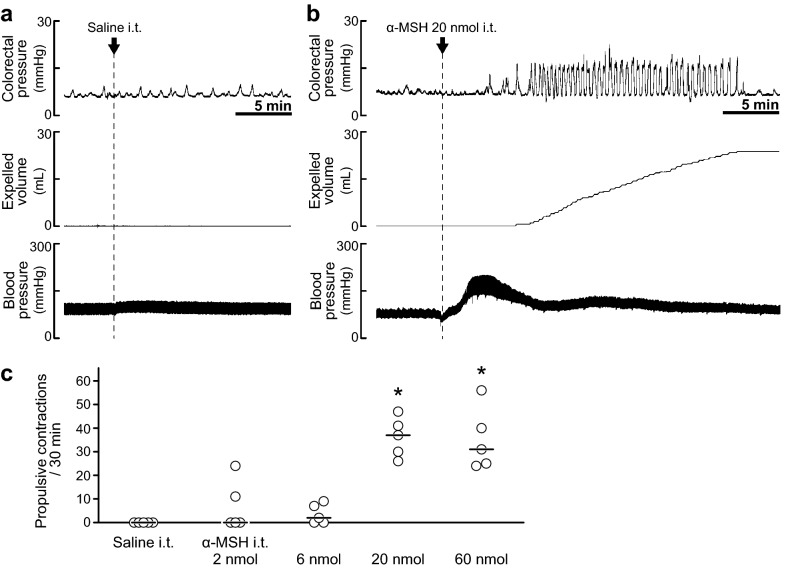
Effects of intrathecal administration of α-MSH at the L6-S1 spinal cord. (**a**,**b**) Typical traces of colorectal pressure (upper), expelled volume (middle) and blood pressure (lower) before and after intrathecal administration (i.t.) of saline (**a**) or α-MSH (20 nmol) (**b**). Although saline had no effect on colorectal motility, α-MSH caused propulsive contractions (transient rises of colorectal pressure accompanied by an increase of expelled volume). (**c**) Summarised graph of number of propulsive contractions during a period of 30 min after saline i.t. or α-MSH i.t. (2, 6, 20 or 60 nmol). Higher doses of α-MSH (20, 60 nmol) significantly increased the number of propulsive contractions compared to that in the saline group. Each open circle (○) indicates a result from an individual rat, and the black bar shows the median. Five rats were used in each group. *Denotes a significant difference from the saline group (*P* < 0.05).

After stabilisation of colorectal motility for 1 h, phasic small increases of colorectal pressure spontaneously occurred, but no evacuation of intracolonic fluid was observed. Under that condition, saline or α-MSH (2, 6, 20 or 60 nmol) was administered into the spinal cord at the L6-S1 level to examine the effects of α-MSH on colorectal motility. Although intrathecal administration of saline did not cause propulsive contractions of the colorectum (Fig. 1a), intrathecal administration of α-MSH caused phasic increases of colorectal pressure accompanied by evacuation of intracolonic fluid (Fig. 1b,c). The numbers of propulsive contractions for 30 min after α-MSH administration were as follows: 2 nmol, 7.0 ± 10.6 (n = 5); 6 nmol, 3.6 ± 4.1 (n = 5); 20 nmol, 36.2 ± 8.4 (n = 5); 60 nmol, 35.2 ± 13.2 (n = 5). Intrathecal administration of α-MSH also increased blood pressure (Fig. 1b). Since α-MSH had a significant effect at higher doses (20 and 60 nmol) but not at lower doses (2 and 6 nmol) (Fig. 1c), we used 20 nmol of α-MSH for intrathecal administration in additional experiments.

**Figure 2:**
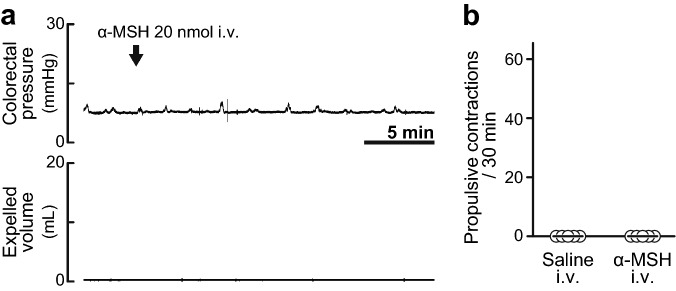
Effects of intravenous injection of α-MSH. (**a**) Typical traces of colorectal pressure (upper) and expelled volume (lower) with intravenous injection (i.v.) of α-MSH (20 nmol). α-MSH i.v. had no effect on colorectal motility. (**b**) Summarised graph of number of propulsive contractions during a period of 30 min after saline i.v. or α-MSH i.v. (20 nmol). There was no significant difference between the saline group and α-MSH group. Each open circle (○) indicates a result from an individual rat, and the black bar shows the median. Five rats were used in each group.

In contrast to intrathecal administration, intravenous administration of α-MSH (20 nmol) did not cause enhancement of colorectal motility (Fig. 2a,b).

### Effect of transection of the thoracic cord on the prokinetic action of α-MSH

**Figure 3:**
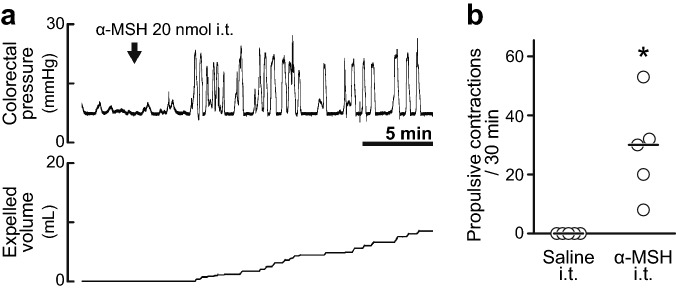
Influence of T4 transection on the α-MSH effect. (**a**) Typical traces of colorectal pressure (upper) and expelled volume (lower) with α-MSH (20 nmol, i.t.) in a rat in which the thoracic spinal cord was transected at the T4 level. α-MSH i.t. caused propulsive contractions. (**b**) Summarised graph of number of propulsive contractions during a period of 30 min after saline i.t. or α-MSH i.t. (20 nmol) in spinalised rats. α-MSH (20 nmol, i.t.) significantly increased the number of propulsive contractions in spinalised rats compared to that in the saline group. Each open circle (○) indicates a result from an individual rat, and the black bar shows the median. *Denotes a significant difference from the saline group (*P* < 0.05). Five rats were used in each group.

To disconnect between the spinal defaecation centre and supraspinal regions, we transected the T4 level thoracic cord. In spinalised rats, spontaneous small contractions of the colorectum were observed in a manner similar to that in intact rats. Although intrathecal administration of saline to the L6-S1 spinal cord had no effect on colorectal motility, α-MSH (20 nmol) caused propulsive contractions even after transection of the thoracic cord (Fig. 3a,b). α-MSH significantly increased the number of propulsive contractions (28.6 ± 16.6, n = 5) compared to the saline administration group (n = 5) (Fig. 3b).

### Effect of transection of the lumbar colonic nerves or the pelvic nerves on the prokinetic action of α-MSH

**Figure 4:**
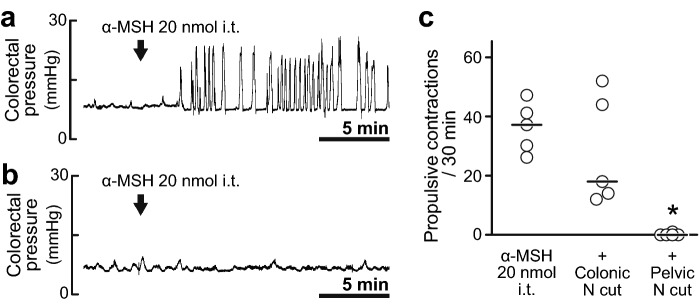
Neural pathways from the spinal defaecation centre to the colorectum. (**a**,**b**) Typical traces of colorectal pressure with α-MSH (20 nmol, i.t) in rats in which the colonic nerves (**a**) or the pelvic nerves (**b**) were severed. Severing the pelvic nerves prevented the prokinetic action of α-MSH (20 nmol), whereas severing the lumbar colonic nerves had no effect. (**c**) Summarised graph of number of propulsive contractions during a period of 30 min after α-MSH i.t. (20 nmol) in control (left), colonic nerve-severed (middle) and pelvic nerve-severed rats (right). The number of propulsive contractions induced by α-MSH (20 nmol, i.t.) in rats in which the pelvic nerves were severed significantly decreased compared to that in the control group. Each open circle (○) indicates a result from an individual rat, and the black bar shows the median. *Denotes a significant difference from the control group (*P* < 0.05). Five rats were used in each group.

The spinal defaecation centre connects to the colorectum through two pathways: the sympathetic lumbar colonic nerves and the parasympathetic pelvic nerves. Each nerve was severed to clarify which pathway relays neural signals evoked by α-MSH in the spinal defaecation centre. In both rats in which the lumbar colonic nerves had been severed and rats in which the pelvic nerves had been severed, spontaneous contractions of the colorectum similar to those in intact rats were observed. In rats in which the lumbar colonic nerves had been severed, intrathecally administered α-MSH (20 nmol) caused propulsive contractions (28.0 ± 18.6, n = 5) (Fig. 4a). In rats in which the pelvic nerves had been severed, α-MSH (20 nmol) failed to cause propulsive contractions of the colorectum (0.2 ± 0.4, n = 5) (Fig. 4b). The number of propulsive contractions induced by intrathecally administered α-MSH was significantly decreased in the rats with pelvic nerve severing but not in rats with lumbar colonic nerve severing compared to that in non-severed rats (Fig. 4c).

### mRNA expression of melanocortin receptors in the spinal defaecation centre

**Figure 5:**
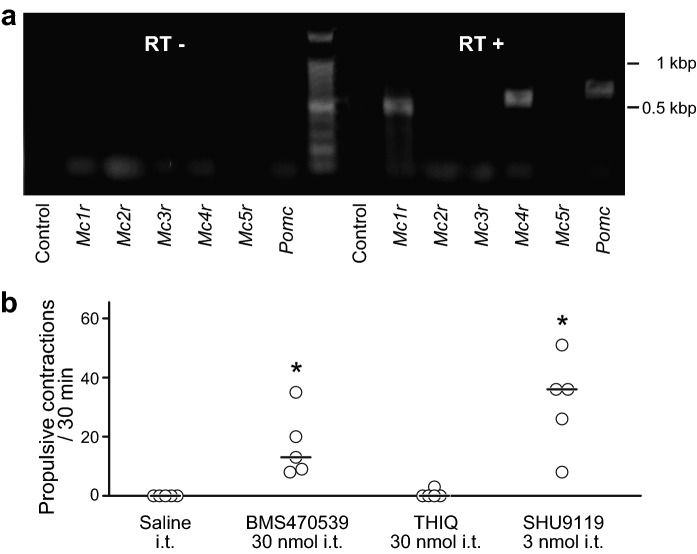
Subtypes of MCR responsible for the α-MSH effect. (**a**) Single-stranded cDNA was generated from total RNA isolated from L6-S1 spinal cord tissue using reverse transcriptase (RT +) and amplified using PCR with primer pairs specific for rat *Mc1r*-*Mc5r* and *Pomc*. Amplified products of *Mc1r*, *Mc4r* and *Pomc* were observed in appropriate sizes. The reactions without reverse transcriptase (RT-) were analysed with PCR as a negative control. (**b**) Summarised graph of number of propulsive contractions during a period of 30 min after i.t. of saline, BMS470539 (MC1R agonist, 30 nmol), THIQ (MC4R agonist, 30 nmol) and SHU9119 (MC1R agonist and MC4R antagonist, 3 nmol). BMS470539 and SHU9119, but not THIQ, significantly increased the number of propulsive contractions compared to that in the saline group. Each open circle (○) indicates a result from an individual rat, and the black bar shows the median. *Denotes a significant difference from the saline group (*P* < 0.05). Five rats were used in each group.

To investigate which subtypes of MCR are expressed in the spinal defaecation centre, we performed RT-PCR analysis of mRNA extracted from the L6-S1 spinal cord using specific primers for melanocortin-1 receptor (MC1R)–MC5R subtypes. Amplified products of *Mc1r* and *Mc4r* were observed in appropriate sizes, whereas transcripts for *Mc2r*, *Mc3r* and *Mc5r* were not detected (Fig. 5a). In addition, the RT-PCR analysis revealed expression of *Pomc*, which is precursor of α-MSH, in the spinal defaecation centre (Fig. 5a).

### Receptor subtypes responsible for α-MSH action in the spinal defaecation centre

To clarify the receptor subtype responsible for α-MSH action in the spinal defaecation centre, pharmacological experiments were performed using the MC1R-selective agonist BMS470539^29^ or the MC4R-selective agonist THIQ^30^. Although intrathecally administered THIQ (30 nmol) at the L6-S1 level failed to enhance colorectal motility (0.6 ± 1.3, n = 5) (Fig. 5b), intrathecally administered BMS470539 (30 nmol) caused propulsive contractions of the colorectum (17.0 ± 11.1, n = 5) (Fig. 5b). To confirm the results, SHU9119, which acts as an agonist for MC1R and an antagonist for MC4R^31^, was intrathecally administered at the L6-S1 level. SHU9119 (3 nmol) caused propulsive contractions of the colorectum (29.4 ± 17.25, n = 5) (Fig. 5b).

## Discussion

In the present study, we aimed to determine the possible involvement of α-MSH in regulation of the function of the spinal defaecation centre in rats. Our major findings are (1) α-MSH administered to the spinal cord at the L6-S1 level caused propulsive contractions of the colorectum in anaesthetised rats with ketamine and α-chloralose, (2) severing the pelvic nerves, but not severing the lumbar colonic nerves or T4 thoracic transection, abolished the effect of intrathecally administered α-MSH, (3) MC1R mRNA and MC4R mRNA were expressed in the spinal cord at the L6-S1 level, and (4) intrathecally administered MC1R, but not MC4R, agonists enhanced colorectal motility. These findings demonstrate that intrathecally administered α-MSH at the L6-S1 level acts on MC1R in the spinal defaecation centre and subsequently activates the pelvic nerves, leading to enhancement of colorectal motility in anaesthetised rats. To our knowledge, this is the first study showing the functional role of α-MSH in the spinal defeacation centre. This study provides new insights into mechanisms by which defaecation is regulated by the central nervous system and pathophysiology of defaecation disorders.

Our results demonstrate that α-MSH enhances colorectal motility when administered intrathecally to the L6-S1 spinal cord by activating the spinal defaecation centre. The finding that α-MSH and ghrelin share common prokinetic actions in the spinal defaecation centre is interesting because α-MSH exerts an opposite effect to that of ghrelin in the hypothalamus. Considering the expression of MCR in the gastrointestinal tract^32^, one possible explanation for the prokinetic effects of α-MSH is that intrathecally administered α-MSH leaks into circulation and activates these receptors in the periphery. However, this is not the case because intravenous injection of α-MSH had no effect on colorectal motility (Fig. 2). Alternatively, the fact that MCR is abundantly expressed in various brain regions should be taken into consideration when evaluating the action site of intrathecally administered α-MSH. The dorsal motor nucleus of the vagus, an important region regulating gastrointestinal motility, expresses MC4R^26^. Since intrathecally administered α-MSH is accessible to the brain region through cerebrospinal fluid flow, contribution of these receptors to the enhancement of colorectal motility is possible. We found that even after disconnection between the supraspinal regions and the spinal defaecation centre by transection of the T4 level thoracic cord, the prokinetic effects of α-MSH remained unchanged (Fig. 3). Taken together, it is reasonable to propose that the action site of intrathecally administered α-MSH is the L6-S1 spinal cord, in which the spinal defaecation centre is located.

Melanocortin receptors are classified into 5 subtypes, MC1R–MC5R. α-MSH binds to MC1R, MC3R, MC4R and MC5R, all of which are usually coupled to Gs proteins and increase intracellular cAMP^33^. In general, major MCR subtypes expressed in the central nervous system are MC3R and MC4R^34^. In the spinal cord, mRNA for MC4R, but not that for MC3R, was detected^27^. In line with this, we also detected MC4R mRNA in the spinal cord at the L6-S1 level (Fig. 5a). On the other hand, MC1R is known to be expressed on skin melanocytes and hair follicles regulating pigmentation^15^. Expression of MC1R has not been reported in the central nervous system except for the periaqueductal grey matter^35^. Therefore, our results showed for the first time, to our knowledge, expression of MC1R, at least at the mRNA level, in the spinal cord.

While two types of melanocortin receptor have been identified in the spinal defaecation centre, MC1R would be responsible for the prokinetic action of α-MSH. Since the conclusion is largely based on results of pharmacological experiments, receptor specificity of drugs should be taken into consideration. In this study, we used BMS470539 as an agonist for MC1R. The specificity of BMS470539 for human MC1R has been reported (EC50 for human MC1R: 28 ± 12 nM, EC50 for human MC4R: 2600 ± 200 nM)^29^, but that for rat MC1R has not been verified. THIQ, used as an agonist for rat MC4R, is thought to be effective for activating rat MC4R (EC50 for rat MC4R: 2.9 ± 0.8 nM)^30^, though data on EC50 of the drug for rat MC1R is not available. Despite these limitations, it is likely that MC1R is the receptor responsible for the effect of α-MSH. SHU9119, used as an agonist for MC1R, has an antagonistic effect for rat MC4R (Ki for rat MC4R: 0.238 ± 0.060 nM)^36^. Considering that only MC1R and MC4R are expressed in the spinal cord of rats (Fig. 5a), the fact that SHU9119 enhanced colorectal motility (Fig. 5b) strongly suggests that α-MSH enhances colorectal motility through acting on MC1R in the spinal defaecation centre in rats.

The prokinetic action of α-MSH is mediated by the parasympathetic pelvic nerve in the spinal defaecation centre. In the spinal defaecation centre, intrathecally administered α-MSH could activate two pathways: (1) ascending pathways to supraspinal regions and/or (2) spinal outflow to the colorectum. However, the ascending pathways are unlikely to be an essential route involved in the effects of α-MSH because disconnection between the spinal defaecation centre and supraspinal regions did not prevent the action of α-MSH (Fig. 3). On the other hand, experiments in which neural pathways were severed (Fig. 4) revealed that α-MSH acting at the L6-S1 spinal cord enhances colorectal motility through mediation of the parasympathetic pelvic nerves. At present, it is not clear whether α-MSH activates the preganglionic neurons directly or indirectly. Since we showed the presence of MC1R in the spinal cord simply by RT-PCR analysis using RNA isolated from spinal cord tissue, there is no information about types of cells expressing MC1R in the spinal cord. In order to determine the precise mechanisms of α-MSH action in the spinal defaecation centre, further experiments including morphological and electrophysiological studies are necessary.

α-MSH is a neuropeptide processed from the precursor polypeptide POMC. We detected mRNA of POMC in the L6-S1 spinal cord (Fig. 5a), in agreement with previous reports^27^. Nevertheless, cell bodies containing ACTH or α-MSH were not detected by immunohistochemistry in the spinal cord^23–25^. Therefore, it seems reasonable to assume that supraspinal regions are physiological sources of α-MSH in the spinal cord. In the brain, POMC is mainly expressed in the nucleus tractus solitarius of the brainstem and ARC of the hypothalamus^15,16^. It has been reported that POMC-containing neurons of the ARC project to the spinal cord, especially regions of the intermediolateral cell column (IML) where cell bodies of preganglionic neurons of autonomic efferents are located^23–25^. These facts suggest that the endogenous source of α-MSH in the spinal defaecation centre is POMC-containing neurons of the ARC.

It is well known that stress induces abnormal enhancement of colorectal motility and causes diarrhoea. Our findings might provide a novel theory about the mechanisms of stress-induced diarrhoea. It has been reported that acute stress activates neurons of the PVN in the hypothalamus and enhances colorectal motility^37,38^. CRF-containing PVN neurons project to NPY-containing neurons in the ARC and inhibit neuronal activity of the NPY-containing neurons^39^. Since NPY-containing neurons inhibit POMC-containing neurons in the ARC through GABA release^40,41^, acute stress could lead to disinhibition of the POMC neurons. In fact, it has been shown that acute stress increases expression of POMC mRNA and c-fos mRNA in POMC-containing neurons of the ARC^42,43^. Based on those results together with our results, we propose a novel theory that acute stress-activated POMC-containing neurons of the ARC secrete α-MSH in the spinal defaecation centre, leading to abnormal enhancement of colorectal motility and resulting diarrhoea. In line with our hypothesis, it has been shown that acute stress activated neurons of the IML in the L6-S1 spinal cord and that severing pelvic nerves suppressed stress-induced defaecation^38,44^.

In summary, we have shown colokinetic action of α-MSH in the spinal defaecation centre. Pharmacological experiments showed that MC1R is the receptor responsible for the α-MSH action. Furthermore, to our knowledge, this is the first report about the expression and function of MC1R in the spinal cord. Our findings provide new insights into the physiological mechanisms of the relationship between feeding and defaecation and also into pathophysiological mechanisms of defaecation disorders. Our findings suggest that α-MSH is a candidate for a neurotransmitter from supraspinal regions to the spinal defaecation centre.

## Methods

### Animals

Male Sprague–Dawley rats were purchased from KBT Oriental Co., Ltd. (Saga, Japan). The rats were maintained in plastic cages at 23 °C with a 12:12 h light: dark cycle (lights on at 07:00–19:00 h) and given free access to laboratory chow (CE-2, CLEA Japan, Inc., Tokyo, Japan) and water. The experimental procedures were approved by the president of Gifu University after being reviewed by the Animal Care and Use Committee of Gifu University (permission numbers: H30-178) and by the president of Kagoshima University after being reviewed by the Institutional Animal Care and Use Committee of Kagoshima University (permission numbers: VM17049). The laboratory animal care and program were conducted in accordance with the AAALAC International-approved program in the Experimental Animal Center of Kagoshima University.

### Recording of colorectal motility

Procedures for recording rat colorectal motility were previously described elsewhere^13,45^. Briefly, rats (250–400 g) were anaesthetised with ketamine hydrochloride (50 mg/kg, intramuscular injection) and α-chloralose (60 mg/kg, into the tail vein). Catheters were inserted into the femoral artery and bladder to infuse anaesthetics (ketamine hydrochloride, 3–5 mg/kg/h; α-chloralose, 10–20 mg/kg/h) and to evacuate the bladder, respectively. To measure changes of blood pressure, the arterial catheter was connected to a pressure transducer. The colorectum was cannulated at the distal colon and anus and then filled with warm saline from a Mariotte bottle connected to the distal colon. Intraluminal pressure was maintained at 5–7 mmHg. The anal cannula was connected to a pressure transducer and a fluid outlet through a one-way valve to measure intraluminal pressure and expelled fluid volume, respectively. During experiments, the rats were warmed with a heating pad to maintain their body temperature. After the surgical operation, colorectal motility was recorded for 1 h to stabilise spontaneous contractions of the colorectum.

### Surgical cutting of nerves

In some series of experiments, the pelvic nerves, lumbar colonic nerves or thoracic spinal cord were severed. The pelvic nerves were bilaterally cut at 2 mm from the major pelvic ganglion^14^. The lumbar colonic nerves running along the mesenteric artery were cut at 2 mm from the aorta^46^. The thoracic spinal cord was transected at the T4 spinal level and ligations were performed to prevent leakage of cerebrospinal fluid^3^.

### Administration of drugs

To intrathecally administer drugs, a catheter with a 30 G needle was inserted at the L6-S1 spinal region, in which the spinal defaecation centre is located^47^, after all surgical operation. The insertion of needle had no effect on colorectal motility. Ten µL saline was flushed after 10 µL drug administration. For intravenous administration, a catheter (PE50, Intramedic Clay Adams, Franklin Lakes, NJ, USA) was inserted into the femoral vein, and 0.3 mL saline was flushed after administration of 10 µL α-MSH.

### RNA isolation and reverse transcription-polymerase chain reaction (RT-PCR)

Total RNA was extracted from a tissue homogenate of the rat spinal cord at L6-S1 levels using NucleoSpin RNA (Macherey–Nagel GmbH & Co. KG, Düren, Germany). First-strand cDNA was synthesised from 0.2 µg of total RNA by using a PrimerScript II 1st Strand cDNA Synthesis Kit with Oligo dT Primer (Takara Bio Inc., Shiga, Japan). To exclude the possibility of genomic DNA contamination, a control solution was prepared in the same way as that for the first-strand cDNA synthesis without PrimerScript II RTase. PCR was performed with Tks Gflex DNA Polymerase (Takara Bio Inc., Shiga, Japan). The primer sets (Fasmac Co., Ltd., Kanagawa, Japan) were as follows: *Mc1r* sense 5′-ATGTATTACTTCATCTGCTGTCTGG-3′ and 5′-ATAATGAGGATGAGGAAGAGGTTGA-3′ (predicted size = 647 bp); *Mc2r* sense 5′-AACTCTGATTGTCCTGATGTAGTTG-3′ and 5′-TCATTAAGAGAACATGGAGCACAAA-3′ (predicted size = 676 bp); *Mc3r* sense 5′-GGACAACATCTTCGACTCTATGATC-3′ and 5′-ATGACAGAGTTGCACATGATGAGAA-3′ (predicted size = 534 bp); *Mc4r* sense 5′-GGCTTCACATTAAGAGGATCGCT-3′ and 5′-TTTATGGAACTCCATAGCGCCC-3′ (predicted size = 594 bp)^48^; *Mc5r* sense 5′-CACTCACCTATGTACTTCTTTGTGG-3′ and 5′-ACGGAATTGCACATGATCAGTATAA-3′ (predicted size = 665 bp); *Pomc* sense 5′-GAGATTCTGCTACAGTCGCTC-3′ and 5′-TTGATGATGGCGTTCTTGAA-3′ (predicted size = 678 bp)^49^. The primers for MC1, 2, 3 and 5R were designed by using BLAST (http://blast.ncbi.nlm.nih.gov/Blast.cgi) and ApE software (http://biologylabs.utah.edu/jorgensen/wayned/ape/). Amplifications were performed by 35 cycles. The reaction products were electrophoresed on 1% agarose gels with ethidium bromide (0.2 µg/ml) containing TAE buffer. The gels were exposed to UV light with a UV transilluminator (Limited-Stage II, AMZ system Science, Osaka, Japan).

### Reagents

The following compounds were used: α-chloralose (Fujifilm Wako Pure Chemical Corp., Osaka, Japan), ketamine hydrochloride (Daiichi Sankyo Co., Ltd., Tokyo, Japan), α-MSH (Peptide Institute. Inc., Osaka, Japan), BMS470539 dihydrochloride (Tocris Bioscience, Bristol, UK), THIQ (Santa Cruz Biotechnology, Inc., TX, USA) and SHU9119 (Abcam, Cambridge, UK). α-Chloralose was solubilised with 10% 2-hydroxypropyl-beta-cyclodextrin (Fujifilm Wako Pure Chemical Corp., Osaka, Japan) and then made up with 0.9% saline for infusion. Other reagents were dissolved in distilled water and stored at -20 °C until usage.

### Presentation of data

Colorectal responses to drugs were quantified using data obtained for a period of 30 min after administration of drugs. Data are presented as means ± SD. Statistical analyses were performed by the Steel–Dwass test for two groups or the Steel test for multiple comparisons using Microsoft Excel with Statcel4 add-in (Statcel-the Useful Addin Forms on Excel-4th ed., OMS Ltd., Saitama, Japan). P-values < 0.05 were considered to be statistically significant.

## Supplementary information

Supplementary Information.

## References

[1] Spencer NJ, Hu H (2020). Enteric nervous system: sensory transduction, neural circuits and gastrointestinal motility. Nat. Rev. Gastroenterol. Hepatol..

[2] Sanger GJ, Furness JB (2016). Ghrelin and motilin receptors as drug targets for gastrointestinal disorders. Nat. Rev. Gastroenterol. Hepatol..

[3] Naitou K (2015). Colokinetic effect of noradrenaline in the spinal defecation center: Implication for motility disorders. Sci. Rep..

[4] Naitou K (2016). Stimulation of dopamine D2-like receptors in the lumbosacral defaecation centre causes propulsive colorectal contractions in rats. J. Physiol..

[5] Nakamori H (2018). Exogenous serotonin regulates colorectal motility via the 5-HT2 and 5-HT3 receptors in the spinal cord of rats. Neurogastroenterol. Motil..

[6] Nakamori H (2018). Medullary raphe nuclei activate the lumbosacral defecation center through the descending serotonergic pathway to regulate colorectal motility in rats. Am. J. Physiol. Gastrointest. Liver Physiol..

[7] Naitou K (2018). Descending monoaminergic pathways projecting to the spinal defecation center enhance colorectal motility in rats. Am. J. Physiol. Gastrointest. Liver Physiol..

[8] Nakamori H (2019). Roles of the noradrenergic nucleus locus coeruleus and dopaminergic nucleus A11 region as supraspinal defecation centers in rats. Am. J. Physiol. Gastrointest. Liver Physiol..

[9] Mönnikes H, Schmidt BG, Tebbe J, Bauer C, Taché Y (1994). Microinfusion of corticotropin releasing factor into the locus coeruleus/subcoeruleus nuclei stimulates colonic motor function in rats. Brain Res..

[10] Mönnikes H, Tebbe J, Bauer C, Grote C, Arnold R (2000). Neuropeptide Y in the paraventricular nucleus of the hypothalamus stimulates colonic transit by peripheral cholinergic and central CRF pathways. Neurogastroenterol. Motil..

[11] Naitou K, Shiina T, Sugita R, Nakamori H, Shimizu Y (2015). Characterization of ghrelin-sensitive neurons in the lumbosacral defecation center in rats. Neurogastroenterol. Motil..

[12] Naitou K (2018). Colokinetic effect of somatostatin in the spinal defecation center in rats. J. Physiol. Sci..

[13] Shimizu Y (2006). Evidence that stimulation of ghrelin receptors in the spinal cord initiates propulsive activity in the colon of the rat. J. Physiol..

[14] Hirayama H (2010). Contrasting effects of ghrelin and des-acyl ghrelin on the lumbo-sacral defecation center and regulation of colorectal motility in rats. Neurogastroenterol. Motil..

[15] Anderson EJP (2016). Regulation of feeding and energy homeostasis by α-MSH. J. Mol. Endocrinol..

[16] Padilla SL, Reef D, Zeltser LM (2012). Defining POMC neurons using transgenic reagents: Impact of transient Pomc expression in diverse immature neuronal populations. Endocrinology.

[17] Toda C, Fellow P (2014). Mitochondrial UCP2 in the central regulation of metabolism. Best Pract. Res. Clin. Endocrinol. Metab..

[18] Willesen MG, Kristensen P, Rømer J (1999). Co-localization of growth hormone secretagogue receptor and NPY mRNA in the arcuate nucleus of the rat. Neuroendocrinology.

[19] Nakazato M (2001). A role for ghrelin in the central regulation of feeding. Nature.

[20] Hu J, Jiang L, Low MJ, Rui L (2014). Glucose rapidly induces different forms of excitatory synaptic plasticity in hypothalamic POMC neurons. PLoS ONE.

[21] Qiu J (2014). Insulin excites anorexigenic proopiomelanocortin neurons via activation of canonical transient receptor potential channels. Cell Metab..

[22] Zhan C (2013). Acute and long-term suppression of feeding behavior by POMC neurons in the brainstem and hypothalamus, respectively. J. Neurosci..

[23] Tsou K, Khachaturian H, Akil H, Watson SJ (1986). Immunocytochemical localization of pro-opiomelanocortin-derived peptides in the adult rat spinal cord. Brain Res..

[24] Elias CF (1998). Leptin activates hypothalamic CART neurons projecting to the spinal cord. Neuron.

[25] Iwasa M, Kawabe K, Sapru HN (2013). Activation of melanocortin receptors in the intermediolateral cell column of the upper thoracic cord elicits tachycardia in the rat. Am. J. Physiol. Hear. Circ. Physiol..

[26] Mountjoy KG, Mortrud MT, Low MJ, Simerly RB, Cone RD (1994). Localization of the melanocortin-4 receptor (MC4-R) in neuroendocrine and autonomic control circuits in the brain. Mol. Endocrinol..

[27] Van Der Kraan M (1999). Expression of melanocortin receptors and pro-opiomelanocortin in the rat spinal cord in relation to neurotrophic effects of melanocortins. Mol. Brain Res..

[28] Kishi T (2003). Expression of melanocortin 4 receptor mRNA in the central nervous system of the rat. J. Comp. Neurol..

[29] Herpin TF (2003). Discovery of tyrosine-based potent and selective melanocortin-1 receptor small-molecule agonists with anti-inflammatory properties. J. Med. Chem..

[30] Sebhat IK (2002). Design and pharmacology of N-[(3R)-1,2,3,4-tetrahydroisoquinolinium-3-ylcarbonyl]-(1R)-1-(4-chlorobenzyl)-2-[4-cyclohexyl-4-(1H–1,2,4-triazol-1-ylmethyl) piperidin-1-yl]-2-oxoethylamine (1), a potent, selective, melanocortin subtype-4 receptor agonist. J. Med. Chem..

[31] Hruby VJ (1995). Cyclic lactam α-melanotropin analogues of Ac-Nle4-cyclo[Asp5, D-Phe7, Lys10] α-melanocyte-stimulating hormone-(4–10)-NH2 with bulky aromatic amino acids at position 7 show high antagonist potency and selectivity at specific melanocortin receptors. J. Med. Chem..

[32] Panaro BL (2014). The melanocortin-4 receptor is expressed in enteroendocrine l cells and regulates the release of peptide YY and glucagon-like peptide 1 in vivo. Cell Metab..

[33] Rodrigues AR, Almeida H, Gouveia AM (2015). Intracellular signaling mechanisms of the melanocortin receptors: current state of the art. Cell. Mol. Life Sci..

[34] Cone RD (2006). Studies on the physiological functions of the melanocortin system. Endocr. Rev..

[35] Xia Y, Wikberg JES, Chhajlani V (1995). Expression of melanocortin 1 receptor in periaqueductal gray matter. NeuroReport.

[36] Adan RAH (1999). Characterization of melanocortin receptor ligands on cloned brain melanocortin receptors and on grooming behavior in the rat. Eur. J. Pharmacol..

[37] Bonaz B, Taché Y (1994). Water-avoidance stress-induced c-fos expression in the rat brain and stimulation of fecal output: role of corticotropin-releasing factor. Brain Res..

[38] Million M, Wang L, Martinez V, Taché Y (2000). Differential Fos expression in the paraventricular nucleus of the hypothalamus, sacral parasympathetic nucleus and colonic motor response to water avoidance stress in Fischer and Lewis rats. Brain Res..

[39] Kuperman Y (2016). CRFR1 in AgRP neurons modulates sympathetic nervous system activity to adapt to cold stress and fasting. Cell Metab..

[40] Cowley MA (2001). Leptin activates anorexigenic POMC neurons through a neural network in the arcuate nucleus. Nature.

[41] Horvath TL, Bechmann I, Naftolin F, Kalra SP, Leranth C (1997). Heterogeneity in the neuropeptide Y-containing neurons of the rat arcuate nucleus: GABAergic and non-GABAergic subpopulations. Brain Res..

[42] Larsen PJ, Mau SE (1994). Effect of acute stress on the expression of hypothalamic messenger ribonucleic acids encoding the endogenous opioid precursors preproenkephalin A and proopiomelanocortin. Peptides.

[43] Liu J (2007). The melanocortinergic pathway is rapidly recruited by emotional stress and contributes to stress-induced anorexia and anxiety-like behavior. Endocrinology.

[44] Suda K, Setoyama H, Nanno M, Matsumoto S, Kawai M (2013). Involvement of parasympathetic pelvic efferent pathway in psychological stress-induced defecation. World J. Gastroenterol..

[45] Bogeski G, Shafton AD, Kitchener PD, Ferens DM, Furness JB (2005). A quantitative approach to recording peristaltic activity from segments of rat small intestine in vivo. Neurogastroenterol. Motil..

[46] Takaki M, Neya T, Nakayama S (1980). Sympathetic activity in the recto-rectal reflex of the guinea pig. Pflügers Arch. Eur. J. Physiol..

[47] Vizzard MA, Brisson M, de Groat WC (2000). Transneuronal labeling of neurons in the adult rat central nervous system following inoculation of pseudorabies virus into the colon. Cell Tissue Res..

[48] Caruso C (2007). Activation of melanocortin 4 receptors reduces the inflammatory response and prevents apoptosis induced by lipopolysaccharide and interferon-γ in astrocytes. Endocrinology.

[49] Chu SC (2014). Involvement of hypothalamic PI3K-STAT3 signalling in regulating appetite suppression mediated by amphetamine. Br. J. Pharmacol..

