# Supplementary material for: α-MSH-induced activation of spinal MC1R but not MC4R enhances colorectal motility in anaesthetised rats
Source: Sci Rep. 2021 Jan 12;11:487. doi: 10.1038/s41598-020-80020-x (PMC7803980; doi:10.1038/s41598-020-80020-x)

**Title:**

α-MSH-induced activation of spinal MC1R but not MC4R enhances colorectal motility in anaesthetised rats.

**Authors:**

Hiromi H. Ueda^1,+^, Kiyotada Naitou^1,+^, Hiroyuki Nakamori^2^, Kazuhiro Horii^3^, Takahiko Shiina^3^, Tatsunori Masatani^4^, Mitsuya Shiraishi^1^, Yasutake Shimizu^3,5,*^.

*corresponding

+these authors contributed equally to this work

**Affiliation:**

1. Department of Basic Veterinary Science, Joint Faculty of Veterinary Medicine, Kagoshima University, 1-21-24 Korimoto, Kagoshima 890-0065, Japan.

2. Department of Cell Physiology, Nagoya City University Graduate School of Medical Sciences, 1 Kawasumi, Mizuho-cho, Mizuho-ku, Nagoya 467-8601, Japan.

3. Laboratory of Physiology, Department of Basic Veterinary Science, The United Graduate School of Veterinary Sciences, Gifu University, 1-1 Yanagido, Gifu 501-1193, Japan.

4. Transboundary Animal Diseases Research Center, Joint Faculty of Veterinary Medicine, Kagoshima University, Kagoshima 890-0065, Japan.

5. Center for Highly Advanced Integration of Nano and Life Sciences, Gifu University (G-CHAIN), Gifu, Japan.

**Supplementary figure**


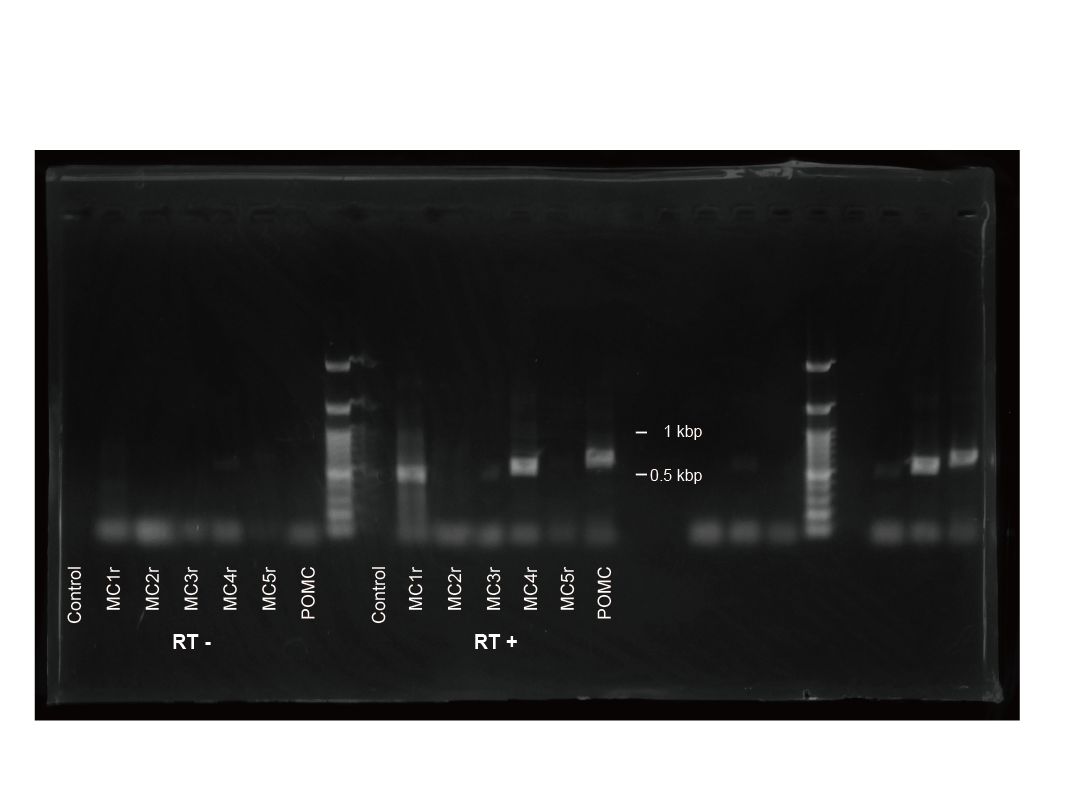

Supplement: Supplementary file 1 — Supplementary Information. [file 41598_2020_80020_MOESM1_ESM.docx]
